# Resolution Enhancement for Millimeter-Wave Radar ROI Image with Bayesian Compressive Sensing

**DOI:** 10.3390/s22155757

**Published:** 2022-08-02

**Authors:** Pengfei Xie, Jianxin Wu, Lei Zhang, Guanyong Wang, Xue Jin

**Affiliations:** 1 School of Electronics and Communication Engineering, Sun Yat-Sen University, Shenzhen 518107, China; xiepf5@mail2.sysu.edu.cn (P.X.); zhanglei57@mail.sysu.edu.cn (L.Z.); 2 Beijing Institute of Radio Measurement, Beijing 100854, China; gywang.sar@gmail.com; 3 Beijing Institute of Tracking and Telecommunication Technology, Beijing 100094, China; cara_snow@163.com

**Keywords:** millimeter-wave imaging, region of interest image, super-resolution, Bayesian compressive sensing

## Abstract

For millimeter-wave (MMW) imaging security systems, the image resolution promisingly determines the performance of suspicious target detection and recognition. Conventional synthetic aperture radar (SAR) imaging algorithms only provide limited resolution in active MMW imaging, which is limited by the system. In terms of enhancing the resolution of a region of interest (ROI) image containing suspicious targets, super-resolution (SR) imaging is adopted via Bayesian compressive sensing (BCS) implemented by fast Fourier transform (FFT). The spatial sparsity of MMW ROI images is well exploited with BCS to achieve resolution enhancement without computational cost. Both simulated and measured experiments confirm that the proposed scheme effectively improves the resolution of ROI images.

## 1. Introduction

Millimeter wave (MMW) signals are widely used in remote sensing, communication, medicine, transportation and other fields. In particular, MMW radar possesses advantages such as high resolution, good penetrability, and an absence of harmful radiation. Thus, it constitutes an alternative for body inspection imaging [1,2,3,4], which is a significant part of MMW security inspection systems. These systems can be sorted into two classes: passive and active. Since the passive millimeter wave radar system is constrained by a non-coherent imaging mechanism, obtaining super-resolution (SR) images through them proves difficult. As a result, we have to adopt image processing algorithms to improve its vision property [5,6,7,8], yielding a radar system with a usual resolution of about several centimeters at the Ka band. By virtue of a synthetic aperture technique, active millimeter-wave radar, on the other hand, achieves up to millimeter-scale resolution at the Ka band, offering advantages in three-dimensional (3D) and high-resolution imaging. As is commonly known, active MMW holographic imaging resolutions are mainly constrained by system parameters, leading to a compromise between the two features when it comes to system design.

As imaging resolution plays a key role in complex security recognition, its enhancement attracts considerable research interest. In order to raise the resolution, most studies have concentrated on improving the architecture of an active MMW system. In [8], a method based on an oversampling technique and a compressive sensing (CS) algorithm is utilized to enhance the resolution of active MMW images. In contrast to whole pixel step scanning, a smaller pixel step is adopted in [8] to increase the sharpness of the image edges. In [9], the structured illumination has been applied in an active MMW image system. Such a structure has the potential to obtain an SR image with up to twofold improvement. In [10], a sparse multi-static one-dimensional antenna array is developed to improve the performance of the cylindrical MMW imaging system. In this way, the number of antennas and the computational cost is reduced significantly, although the quality of the reconstructed image becomes inferior to that produced in full sampling conditions. In [11], besides the adaption of the CS technique, a novel sparse array is developed to reduce the number of antennas and thus raise the peak signal-to-noise ratio (PSNR) of the reconstructed image.

Although these methods [8,9,10,11] are effective to improve the image resolution, they have limitations in acquiring MMW SR images. To begin with, these methods require typically demanding and costly modifications to the imaging system’s structure and parameters. Furthermore, the imaging resolution obtained through these methods’ sparse array structures usually proves much worse than that yielded by full-sample systems. Finally, these methods do not fully utilize the prior information of MMW-3D images. To overcome these shortcomings, this paper proposes a new MMW image SR algorithm based on image sparsity and a CS technique.

Active MMW body inspection systems based on synthetic aperture radar (SAR) imaging theory usually provide sparse images due to the limited penetration of MMW. CS [12,13,14] stands as an effective technique for the SR image reconstruction of these sparse images. More importantly, Bayesian compressive sensing (BCS), an application development of CS in sparse reconstruction, can make full use of the sparse prior knowledge of the signal [15,16], and it proves more suitable for active MMW image SR. Therefore, this paper proposes region of interest (ROI) image SR with BCS in order to exploit the image sparsity and effectively carry out threat detection. From the perspective of target detection and recognition, MMW image SR can be regarded as feature enhancement as well as a method of improving SAR image quality and target recognition performance [17]. This work’s main contribution lies in that it applies the sparsity-driven SR technique to active MMW 3D imagery without any system structure modification. ROI image resolution enhancement with BCS is achieved for suspicious target recognition. A regional 3D MMW image SR scheme is designed to reduce the computational burden of BCS, thus promoting the efficiency of threat detection and recognition.

The remainder of this paper is organized as follows. In Section 2, we detail the 3D holographic imaging algorithm and the wave number spatial spectrum characteristics of the MMW image. In Section 3, the sparsity of the 3D MMW image is analyzed and the ROI image resolution enhancement method based on BCS is achieved. In Section 4, the feasibility and effectiveness of the ROI image SR with BCS are demonstrated by the experiments with simulated data and measured data. Finally, this paper is concluded in Section 5.

## 2. Holographic Imaging and Wave Number Spatial Spectrum

### 2.1. System Model and Holographic Imaging

**Figure 1:**
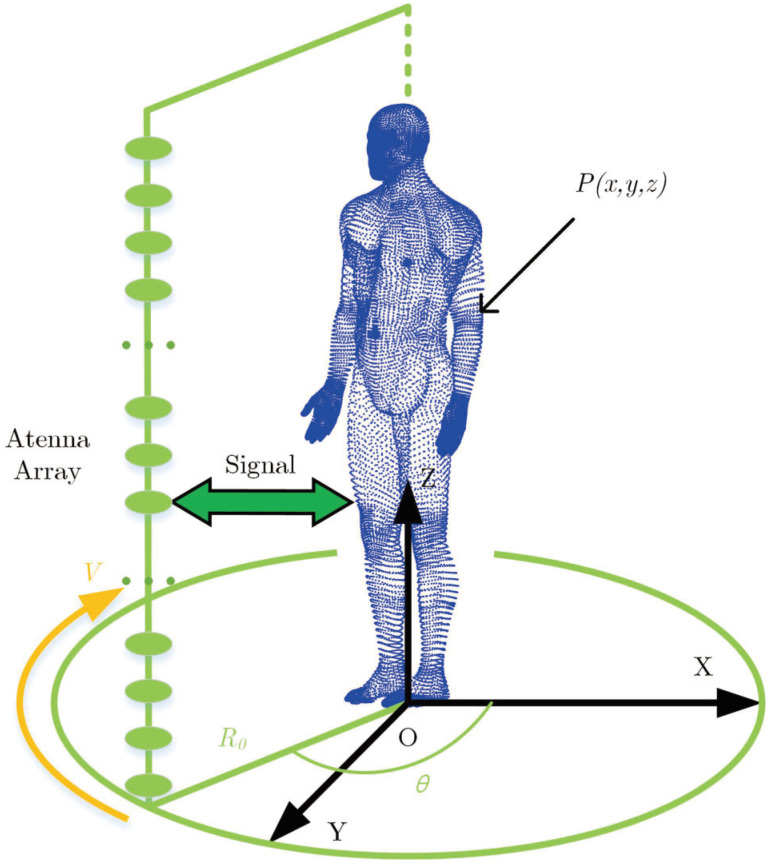
Model of an active MMW 3D holographic imaging system.

In an active MMW 3D holographic imaging system, a set of linear array is used to collect the echo signal in an all-round way by moving along the circular path while alternately scanning, and then, the 3D high-resolution images are obtained by the holographic imaging algorithm. According to SAR imaging theory, high resolution in the range dimension is achieved by transmitting and receiving wideband signals, while high resolution in the array dimension is achieved by synthetic aperture formed by array scanning, and high resolution in the azimuth dimension is achieved by synthetic aperture formed by array motion. The operating mode of the active MMW 3D holographic imaging system is shown in Figure 1.

As shown in Figure 1, the antenna array rotates around the Z axis with the radius *R*${}_{0}$, *V* is the motion direction of the array, θ is the rotation angle of the array, and *P* is a scattering point of the scanning area. In the radar imaging coordinate system, X, Y, and Z correspond to the azimuth dimension, range dimension, and array dimension (height dimension) of the radar, respectively. In an active MMW 3D holographic imaging system, a set of linear array is used to collect the echo signal in a 360 degree pathway while alternately scanning, and then, the 3D high-resolution images are obtained by the holographic imaging algorithm. According to [1], the echo signal received by an antenna element at position $\left(R_{0},\theta ,Z_{h}\right)$ can be written as

(1) $$s\left(f_{r},\theta ,Z_{h}\right)=\;\int \int \int \;\sigma \left(x,y,z\right)\exp\left\{-jK_{r}R_{xyz}\right\}dxdydz$$

(2) $$R_{xyz}=\sqrt{{\left(R_{0}\cos\theta -x\right)}^{2}+{\left(R_{0}\sin\theta -y\right)}^{2}+{\left(Z_{h}-z\right)}^{2}}$$

where $\sigma \left(x,y,z\right)$ is the scattering coefficient of the scattering point at $\left(x,y,z\right)$, and $R_{xyz}$ is the Euclidean distance from the antenna element to the scattering point. $K_{r}=4\pi f_{r}/c$ is the wave number domain representation of fast time frequency $f_{r}$, and *c* is the propagation speed of electromagnetic waves. The Fourier transform of (1) along the Z direction can be written as

(3) $$S\left(K_{r},\theta ,K_{z}\right)=\int s\left(K_{r},\theta ,Z_{h}\right)\exp\left\{-jK_{Z_{h}}Z_{h}\right\}dz$$

where $K_{Z_{h}}$ is the Fourier-transform variable corresponding to $Z_{h}$, Equation (3) is an integral equation, and its phase term is

(4) $$\phi_{sq}\left(K_{r},\theta ,K_{Z_{h}}\right)=-jK_{r}R_{xyz}-jK_{Z_{h}}Z_{h}$$

Here, we rewrite $R_{xyz}$ as $R_{xyz}=\sqrt{{R_{xy}}^{2}+{\left(Z_{h}-z\right)}^{2}}$, where $R_{xy}^{2}={\left(R_{0}\cos\theta -x\right)}^{2}+{\left(R_{0}\sin\theta -y\right)}^{2}$ denotes the projection length of $R_{xyz}$ on the X-Y plane, and the following equation can be obtained from $\frac{\partial }{\partial Z_{h}}\phi_{sq}\left(K_{r},\theta ,K_{z_{h}}\right)=0$

(5) $$K_{r}\frac{Z_{h}-z}{\sqrt{R_{xy}^{2}+{\left(Z_{h}-z\right)}^{2}}}+K_{Z_{h}}=0$$

where $\frac{\partial }{\partial x}\left(\cdot \right)$ represents a partial differential operation on *x*. The solution of (5) is

(6) $$Z_{h}^{*}=-\frac{K_{Z_{h}}}{\sqrt{K_{r}^{2}-K_{Z_{h}}^{2}}}R_{xy}+z$$

According to the principle of stationary phase (POSP) [18], we substitute $Z_{h}^{*}$ in (6) for $Z_{h}$ in (3) and remove the integral sign of the Z-direction. Then, the analytical expression of (3) can be written as (7).

(7) $$S\left(K_{r},\theta ,K_{z}\right)=\;\int \int \int \;\sigma \left(x,y,z\right)\exp\left\{-j\sqrt{K_{r}^{2}-K_{Z_{h}}^{2}}R_{xy}\right\}\exp\left\{-jK_{Z_{h}}z\right\}dxdydz$$

The first phase term in (7) is the coupling phase term, and the signal decoupling of (7) can be carried out by using the omega-K algorithm [19,20]. The variable substitution can be written as

(8) $$\sqrt{K_{r}^{2}-K_{Z_{h}}^{2}}\to K_{rc}+K_{xy}$$

where $K_{rc}=4\pi f_{c}/c$ is the center of the frequency wave number, $f_{c}$ is the center frequency of the signal, and $K_{xy}$ is the wave number distribution on the X-Y plane after the decoupling. Equation (8) represents Stolt Mapping (SM) that is implemented by interpolation. The decoupled signal of (7) is written as (9).

(9) $$S\left(K_{r},\theta ,K_{Z_{h}}\right)=\;\int \int \int \;\sigma \left(x,y,z\right)\exp\left\{-jK_{xy}R_{xy}\right\}\exp\left\{-jK_{Z_{h}}z\right\}\exp\left\{-jK_{rc}R_{xy}\right\}dxdydz$$

Since both $Z_{h}$ and *z* in (9) are variables in the Z dimension, assuming that the window functions are rectangular windows, we can obtain the two-dimensional (2D) inverse Fourier transform of (9) along the frequency and Z dimensions as (10).

(10) $$S\left(t,\theta ,Z_{h}\right)=\mathrm{sinc}\left(t-\tau \right)\mathrm{sinc}\left(Z_{h}-z\right)\int \sigma \left(x,y\right)\exp\left\{-jK_{rc}R_{xy}\left(\theta \right)\right\}d\theta$$

In (10), *t* is the Fourier-transform variable corresponding to $K_{xy}$, and τ is the time delay caused by $R_{xy}$. After the SM in (8), the signals in (10) are not coupled on the Z-X-Y plane. The signals received by the antenna element with the height of $Z_{h}$ can be rewritten as

(11) $$S\left(t,\theta \right)=\mathrm{sinc}\left(t-\tau \right)\int \sigma \left(x,y\right)\exp\left\{-jK_{rc}R_{xy}\left(\theta \right)\right\}d\theta$$

The back-projection (BP) imaging algorithm [21,22] can be used to focus the signals. Thus, the imaging process can be accomplished by

(12) $$\sigma \left(x,y\right)=\int S\left(t,\theta \right)\exp\left\{jK_{rc}R_{xy}\right\}d\theta$$

Since $Z_{h}$ is arbitrary, we operate (12) for all $Z_{h}$ to obtain $\sigma \left(x,y,z\right)$, which means reconstructing a 3D holographic image of the target to focus the signals received by all antenna elements.

**Figure 2:**
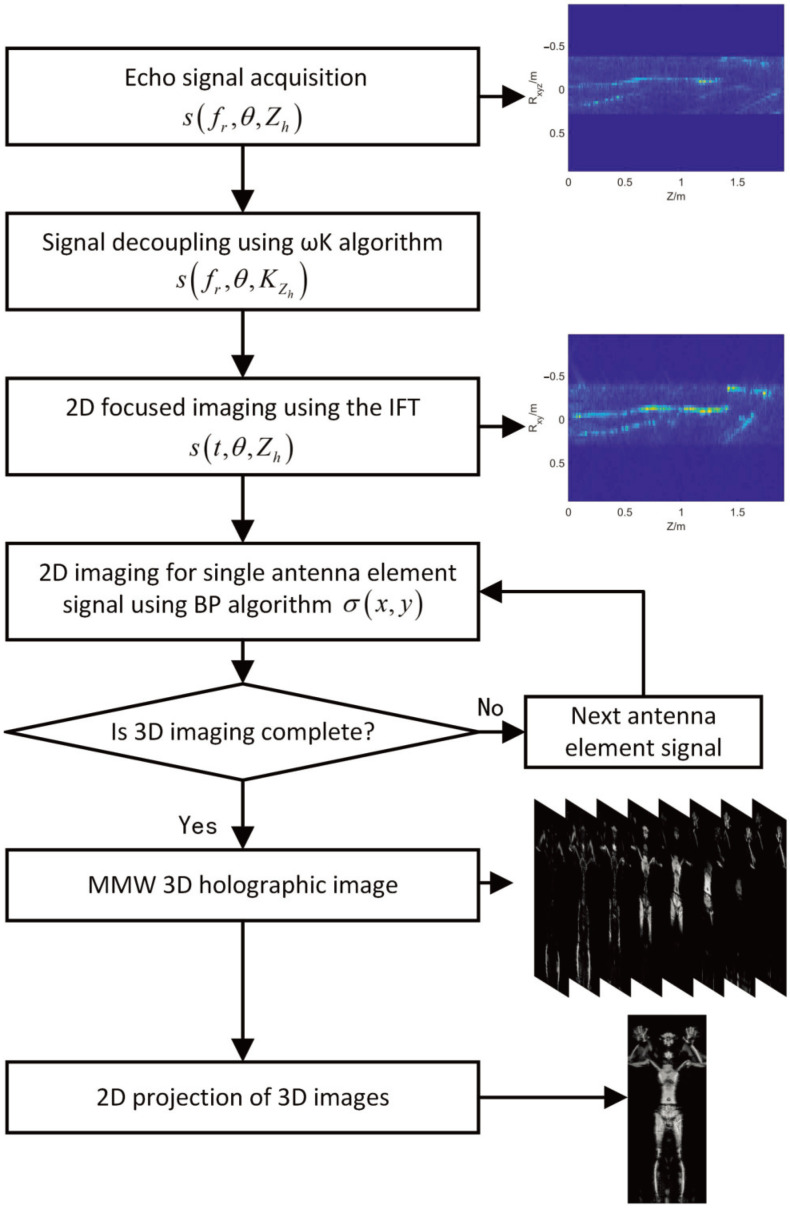
Flowchart of millimeter wave holographic imaging.

Generally, in the process of body inspection, we can project the 3D image of a target on the X–Z plane through maximum projection to obtain its 2D projection image. Assume that the size of the original 3D holographic image along the X, Y and Z directions is $N_{x}$, $N_{y}$ and $N_{z}$, respectively. Then, the 3D image can be regarded as a collection of 2D images with the size of $N_{x}\times N_{z}$. The pixel value of the 2D projected image at $\left(x,z\right)$ is defined as

(13) $${\mathrm{Img}}_{2D}\left(x,z\right)=\max\left\{{\mathrm{Img}}_{3D}\left(x,z,1:N_{y}\right)\right\}$$

where ${\mathrm{Img}}_{3D}$ denotes the 3D holographic image, and ${\mathrm{Img}}_{2D}$ denotes the 2D projection image. The slice index of the maximum pixel in the 2D projection image is defined as the pixel depth of the 2D projection image. According to the foregoing derivation, the process description of holographic imaging is shown in Figure 2.

### 2.2. Spatial Wave Number Spectrum and Resolution

**Figure 3:**
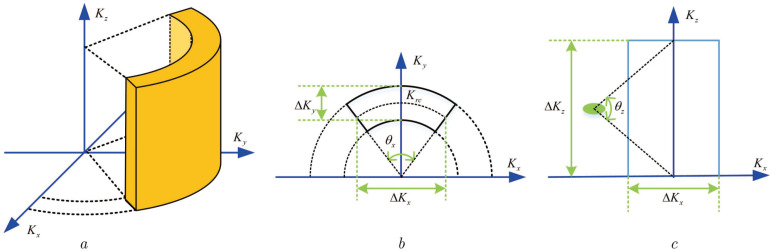
Spatial wave number spectrum distribution of 3D image. (**a**) Schematic diagram of 3D wave number spectrum. (**b**) Wave number spectrum of X-Y plane. (**c**) Wave number spectrum of X-Z plane.

In MMW 3D imaging, the resolution of the image is determined by the coverage of the wave number spectrum (spatial frequency domain). According to the dispersion relationship of plane waves in free space or uniform medium, we have

(14) $$K_{x}^{2}+K_{y}^{2}+K_{z}^{2}=K_{r}^{2}$$

where $K_{x}=K_{xy}\cos\theta$, $K_{y}=K_{xy}\sin\theta$, and $K_{z}=K_{Z_{h}}$ are the components of $K_{r}$ in the X, Y, and Z coordinate systems, respectively. The 3D spatial wave number spectrum distribution is shown in Figure 3.

As shown in Figure 3, Figure 3a is the 3D wave number spectrum distribution of the MMW image. It is like a hollow cylinder. Figure 3b is the wave number spectrum distribution on the X–Y plane. This distribution pattern looks like the bottom area of the hollow cylinder. $\Delta K_{x}$ is the width of the wave number spectrum in the X dimension, $\Delta K_{y}$ is the width of the wave number spectrum in the Y dimension, and $\theta_{x}$ is the backscattering angle of the target. According to the geometric relationship, $\Delta K_{x}$ and $\Delta K_{y}$ can be written as

(15) $$\Delta K_{x}=2K_{rc}\sin\left(\theta_{x}/2\right)$$

(16) $$\Delta K_{y}=4\pi B/c$$

where *B* is the signal bandwidth of the system. Figure 3c shows the wave number spectrum distribution on the X-Z plane. Its pattern is rectangular. $\Delta K_{z}$ is the width of the wave number spectrum in the Z dimension, and $\theta_{z}$ is the beam width of the antenna. $\Delta K_{z}$ can be defined as

(17) $$\Delta K_{z}=2K_{rc}\sin\left(\theta_{z}/2\;\right)$$

For a wideband signal, the relationship between the bandwidth and the pulse width satisfies δ=2π/ΔK, where δ is the pulse width (that is, the spatial resolution) of the signal after pulse compression, and ΔK is the bandwidth of the signal. Furthermore, the resolutions in 3D space can be defined as

(18) $$\delta_{x}=\frac{2\pi }{\Delta K_{x}}=\frac{\lambda }{4\sin\left(\theta_{x}/2\;\right)}$$

(19) $$\delta_{y}=\frac{2\pi }{\Delta K_{y}}=\frac{c}{2B}$$

(20) $$\delta_{z}=\frac{2\pi }{\Delta K_{z}}=\frac{\lambda }{4\sin\left(\theta_{z}/2\;\right)}$$

where $\lambda =c/f_{c}$ denotes the wavelength corresponding to the central frequency of the wideband signal.

In theory, holographic imaging systems can be used to obtain the image with high resolution. However, in real active millimeter-wave imaging systems, the image resolution is degraded due to limitations of signal processing algorithms, such as bandwidth loss. From (18) to (20), we know that the resolution of the system can be increased by adjusting and optimizing system parameters such as signal bandwidth and antenna beam width. This resolution enhancement is realized by upgrading the hardware, leading to more work and cost. Hence, in the threats detection and recognition, it is more economical and convenient to adopt the signal and image processing, instead of upgrading the hardware, for the resolution enhancement of the MMW images.

## 3. Resolution Enhancement with Bayesian Compressive Sensing

### 3.1. MMW 3D Image Sparsity and ROI

**Figure 4:**
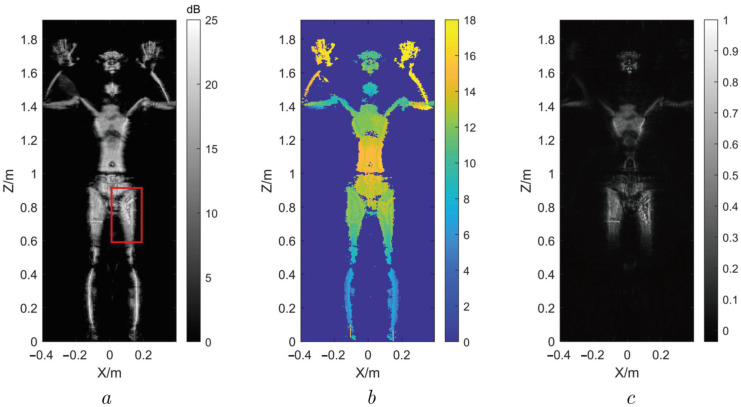
The grayscale distribution and depth distribution of a MMW image. (**a**) Grayscale distribution of a MMW image. (**b**) The pixel depth distribution of a MMW image. (**c**) Normalized grayscale of 12th slice.

Since the MMW imaging of the human body is three-dimensional and the MMW’s penetration is limited, all the 2D slice images of an active MMW 3D holographic image are sparse. Then, we take a 2D projection image for an example to demonstrate this sparsity, as shown in Figure 4. Figure 4a is the grayscale distribution of the 2D projection image of a MMW 3D holographic image obtained by (13), where decibel (dB) is the unit of the pixel value. Figure 4b reflects the distribution of pixel depth. Figure 4c is the normalized grayscale distribution of the 12th slice image of the MMW 3D holographic image. There are different pixel depths indexed by different colors in Figure 4b, which indicates that the 2D slice images of 3D holographic image are sparse. Furthermore, the light and shade in Figure 4c proves the varying of pixel value, indicating the sparsity as well.

In the threat detection through MMW imaging, once the suspicious item is confirmed in the 2D projection image, we extract the part corresponding to the suspicious item (such as the region within the red box in Figure 4a) as the ROI image, and then, the rest of the 2D projection image is considered as the background region. According to this ROI image, we can select the 2D slice images of the MMW 3D holographic image that can reflect the suspicious item and ignore the others. After that, there are fewer 2D slice images that need to be processed so that the computational burden can be greatly reduced.

Therefore, it is necessary to adopt a sparse reconstruction technique for processing the 2D projection image and thus form the ROI image in order to perform threat recognition better and faster.

### 3.2. Bayesian Compressive Sensing

For sparse images, CS is an effective method to solve the sparse reconstruction problem by solving a $l_{1}$ optimization problem. In addition, the prior distribution can be transformed into the posterior distribution through Bayes’ theorem, which is very suitable for our work. Consequently, we apply the BCS algorithm to achieve the super-resolution of sparse images, and a brief derivation of BCS principles will be given as follows.

For a 2D image, the basic model of compressive sensing SAR imaging can be written as (21) [23,24].

(21) R=ΦS+η

where $R={\left[r_{nm}\right]}_{N\times M}$ is a MMW 2D image, $S={\left[s_{\bar{n}m}\right]}_{\bar{N}\times M}$ is the super-resolution image corresponding to R, $\Phi ={\left[\phi_{n\bar{n}}\right]}_{N\times \bar{N}}$ is a compressive sensing matrix, and $\eta \in C^{N\times M}$ represents noise signals. In this paper, R denotes a 2D slice of a 3D image ${\mathrm{Img}}_{3D}$. Taking the image resolution enhancement of the X-Z plane as an example, R can be defined as $R={\mathrm{Img}}_{3D}\left\{:,:,n_{y}\right\}$, where $n_{y}=\left\{1,2,...N_{y}\right\}$ represents the index of the slice image along the Y direction. In this paper, a 2D Fourier transform matrix is selected as the compressive sensing matrix Φ, which is defined as

(22) $$\Phi ={\left[\begin{array}{cccc} 1 & 1 & \cdots & 1\\ 1 & \omega & \cdots & \omega^{\left(\bar{N}-1\right)}\\ \vdots & \vdots & \ddots & \vdots \\ 1 & \omega^{\left(N-1\right)} & \cdots & \omega^{\left(N-1\right)\left(\bar{N}-1\right)} \end{array}\right]}_{N\times \bar{N}}$$

where $\omega =\exp\left\{-j\frac{2\pi }{N}\right\}$. We select the 2D Fourier transform matrix for two reasons. For one, this 2D Fourier matrix operation can be realized by 2D fast Fourier transform (FFT), which can improve the computational efficiency of compressive sensing. In addition, we can obtain the 2D wave number spectrum of an SAR image through this matrix. In fact, according to Section 2.2, the broadening of the wave number spectrum of the MMW image is equivalent to the improvement of the resolution.

According to the definitions of (21) and (22), on the basis of compressive sensing, SR image S can be obtained through estimating the measured image data R. The maximum a posterior (MAP) estimator is

(23) $$\hat{S}\left(R\right)=\underset{S\in C^{\bar{N}\times M}}{\arg\max}\;\left[p\left(S,R\right)\right]$$

In general, the noise term in (21) can be modeled as Gaussian noise with zero mean and the variance of $\sigma^{2}$. Thus, the Gaussian likelihood function of the observed value S is

(24) $$\begin{array}{cc} p\left(R\left|S,\sigma^{2}\right.\right)= & {\left(2\pi \sigma^{2}\right)}^{-N\cdot M}\exp\left\{-\frac{1}{2\sigma^{2}}{∥\eta ∥}_{F}^{2}\right\}\\ = & {\left(2\pi \sigma^{2}\right)}^{-N\cdot M}\exp\left\{-\frac{1}{2\sigma^{2}}{∥R-\Phi S∥}_{F}^{2}\right\} \end{array}$$

According to sparse Bayesian learning [25] and Bayesian compressive sensing [16], the sparsity can be enhanced by Laplacian sparse prior. Assuming that the Laplacian distribution of each pixel of the SR image is independent and identical, the probability density function of the SR image can be written as

(25) $$\begin{array}{cc} p\left(S\left|\gamma \right.\right)= & \prod_{m=0}^{M-1}\prod_{\bar{n}=0}^{\bar{N}-1}\left(\frac{\gamma^{2}}{2\pi }\right)\exp\left\{-\gamma \left|s_{\bar{n}m}\right|\right\}\\ = & {\left(\frac{\gamma^{2}}{2\pi }\right)}^{\bar{N}\cdot M}\exp\left\{-\gamma {∥S∥}_{1}\right\} \end{array}$$

where ${∥S∥}_{1}=\sum_{\bar{n},m}\left|s_{\bar{n}m}\right|$ means the $l_{1}$ norm of S. According to (23) and Bayes rule, we have

(26) $$\begin{array}{cc} \hat{S}\left(R\right)= & \underset{S\in C^{\bar{N}\times M}}{\arg\max}\;\left[p\left(R|S,\sigma^{2}\right)\cdot p\left(S|\gamma \right)\right] \end{array}$$

which is equivalent to (27).

(27) $$\begin{array}{cc} \hat{S}\left(R\right)= & \underset{S\in C^{\bar{N}\times M}}{\arg\max}\;\left\{\log\left[p\left(R|S,\sigma^{2}\right)\cdot p\left(S|\gamma \right)\right]\right\}\\ = & \underset{S\in C^{\bar{N}\times M}}{\arg\min}\;\left\{{∥R-\Phi S∥}_{F}^{2}+\mu {∥S∥}_{1}\right\} \end{array}$$

where $\mu =2\sigma^{2}\gamma$ denotes the sparse coefficient, which is related to the statistical characteristics of the image. To solve (27), a Cauchy–Newton algorithm can be used. In order to make the $l_{1}$ norm in (25) differentiable, the following approximation can be made [26].

(28) $${∥s_{\bar{n}m}∥}_{1}\approx \sqrt{{\left|s_{\bar{n}m}\right|}^{2}+\tau }$$

In order to ensure the accuracy of the value, τ in (28) should be minimized to be less than $10^{-6}$ in practical applications. According to the Cauchy–Newton algorithm [27], the iterative equation to solve (27) can be written as

(29) $${\hat{s}}_{m}^{h+1}={\left[H\left({\hat{s}}_{m}^{h}\right)\right]}^{-1}\Phi^{H}r_{m}$$

where $s_{m}=S\left(m\right)$ and $r_{m}=R\left(m\right)$ represent the *m*th column signals of S and R, respectively, and the superscript *H* means conjugate transpose. $H\left(s_{m}\right)={2\Phi }^{H}\Phi +\mu \cdot U\left(s_{m}\right)$ is the Hessian matrix, and $U\left(s_{m}\right)$ is defined as (30).

(30) $$U\left(s_{m}\right)=\mathrm{diag}\left[\frac{1}{\sqrt{{\left|s_{0m}\right|}^{2}+\tau }},\frac{1}{\sqrt{{\left|s_{1m}\right|}^{2}+\tau }},...,\frac{1}{\sqrt{{\left|s_{\left(\bar{N}-1\right)m}\right|}^{2}+\tau }}\right]$$

From the above derivation, we can know that (29) includes the Hessian matrix inversion. It is worth noting that the conjugate gradient algorithm (CGA) [28], instead of the matrix factorization inversion, is used to solve (29). In addition, according to [27], for each column of signals in (27)

(31) $$f\left(s_{m}\right)={∥r_{m}-\Phi s_{m}∥}^{2}+\mu \left|s_{m}\right|$$

Its conjugate gradient can be expressed as

(32) $$\nabla_{s_{m}}f\left(s_{m}\right)=2\Phi^{H}\Phi s_{m}+\mu U\left(s_{m}\right)s_{m}-\Phi^{H}r_{m}$$

In the iterative process, since the matrix Φ is a Fourier transform matrix, Fourier transform (FFT) can be employed to speed up the computation of $\Phi^{H}\Phi s_{m}$.

Since the compressive sensing algorithm can enhance the resolution of a 2D slice image, a 3D super-resolution image can be obtained by executing the algorithm for each 2D slice image. Finally, according to (13), the 2D image with resolution enhancement can be obtained and used for threat detection and recognition.

### 3.3. Target Region Image Resolution Enhancement

In addition to the effectiveness of BCS, its computational efficiency proves necessary for the MMW image SR. Commonly, CS based on FFT operation can be used to reduce the algorithm complexity of resolution enhancement. However, if we try to enhance the resolution of a whole three-dimensional image in a threat recognition task, the computational load still probably becomes unacceptable.

**Figure 5:**
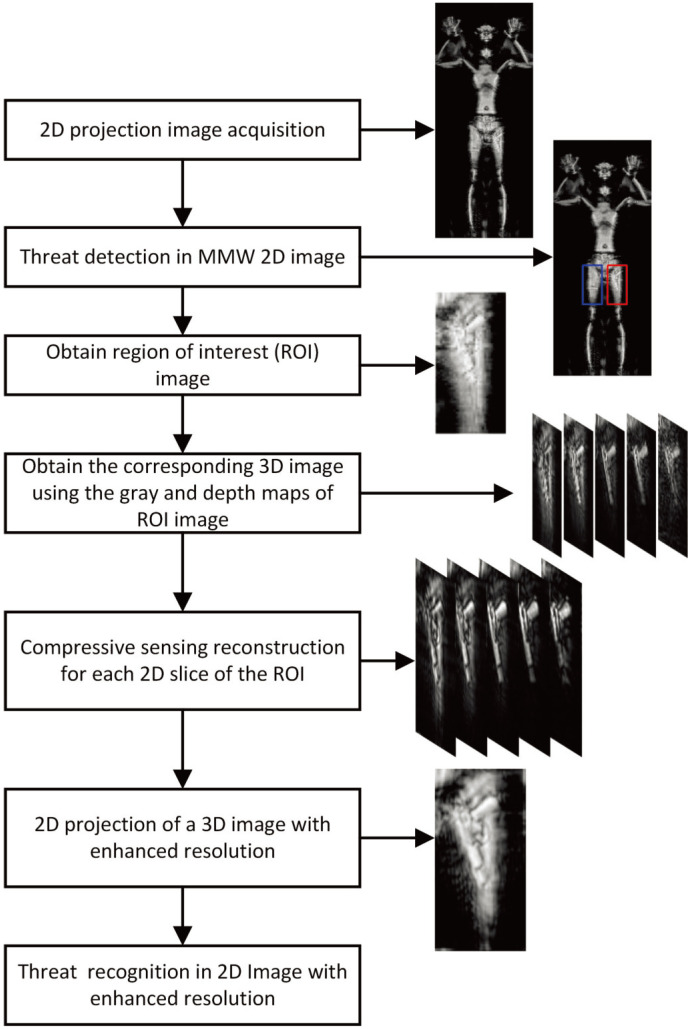
Flowchart of MMW regional image super resolution.

In fact, in a MMW body inspection image, we just need to focus on the part where the suspected target appears instead of the others. As a result, once the suspected target is confirmed in the projected MMW-2D image, the corresponding ROI images can be yielded. According to the projection relation of these ROI images, the 3D image of the suspected target as well as its 2D slice images can be obtained. By the pixel depth, we can choose the slice images that can reflect the suspected target and then enhance the resolution of them through compressive sensing. Based on this idea, a regional 3D imaging SR technique is developed so as to significantly reduce the computational load and raise the accuracy of the object detection and recognition. The flowchart of the regional 3D image SR technique is shown in Figure 5. The regional image SR algorithm not only greatly enhances the resolution of suspected object regions but also inspects them faster.

## 4. Experimental Results and Analysis

### 4.1. Lattice Point Targets Simulation Experiment

**Figure 6:**
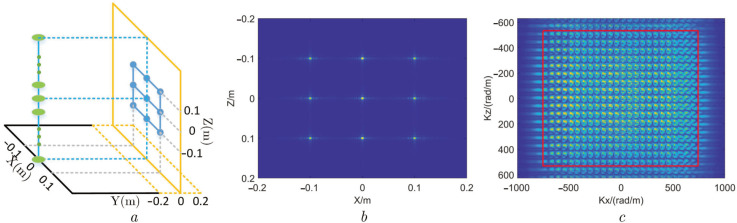
Lattice point targets simulation imaging and 2D wave number spectrum. (**a**) Lattice point targets distribution. (**b**) MMW image of preset targets. (**c**) Two-dimensional (2D) wave number spectrum of image.

To demonstrate the effectiveness and accuracy of the compressive sensing SR algorithm, an echo simulation and 3D imaging experiment of 2D lattice point targets is designed in this section. The operation mode of the MMW 3D imaging system is shown in Figure 1, and the system parameters are shown in Table 1. From Table 1 and (18) to (20), it can be known that the theoretical resolutions of the X dimension, Y dimension, and Z dimension in this simulation experiment are $\delta_{x}=5$ mm, $\delta_{y}=25$ mm, and $\delta_{z}=5$ mm, respectively. The simulation results of 2D lattice point targets imaging are shown in Figure 6.

The image of lattice point targets and its 2D wave number spectrum are shown in Figure 6. Nine point targets have been evenly distributed on the plane y = 0, as shown in Figure 6a. Figure 6b is the MMW image of these targets on the y = 0 m plane. According to Figure 6b, the MMW image of these targets can precisely reflect the distribution of them, indicating that data simulation and imaging in this experiment are reliable. Figure 6c illustrates the 2D wave number spectrum distribution of these targets, which is obtained by performing 2D FFT for the MMW image. In Figure 6c, the 2D wave number spectrum of the MMW image on the X-Z plane presents a rectangular distribution, which has been theoretically discussed in Section 2.2. Finally, the signal in the effective region of the wave number spectrum (the region within the red rectangular in Figure 6c) is selected as the input of the image SR algorithm.

**Figure 7:**
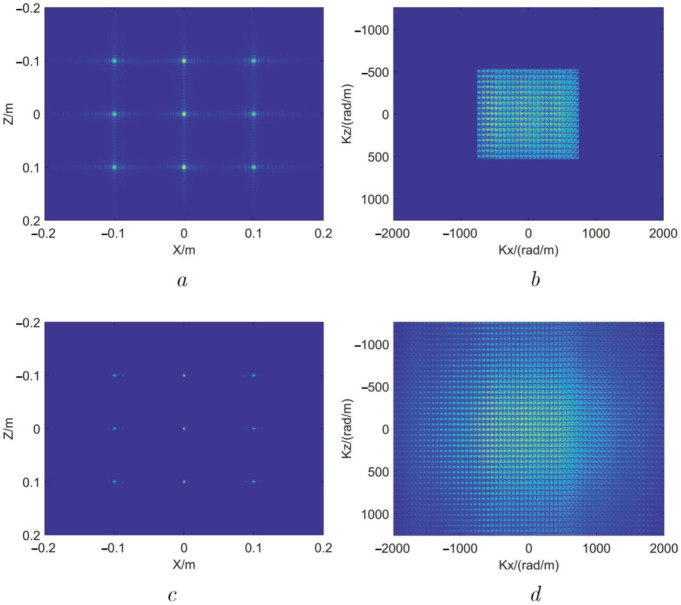
Result of lattice point targets SR experiment. (**a**) MMW image of lattice point targets after padding zeros in wave number domain. (**b**) Wave number spectrum after padding zeros. (**c**) MMW SR image. (**d**) Wave number spectrum of SR image.

In this experiment, the SR image size is set to twice the original image size, and the experimental result is shown in Figure 7.

In order to illustrate the performance of the lattice point targets image SR, the 2D wave number spectrum after the outside of the effective region in Figure 6c is zero-filled, as shown in Figure 7b. We operate a two-dimensional IFFT for the 2D wave number spectrum to its image. After this operation, the image length and width become twice as large as those of the original image, as shown in Figure 7a. Then, we enhance the resolution of the enlarged image through our proposed method and illustrate the result in Figure 7c. The 2D wave number spectrum of the SR image is shown in Figure 7d. From Figure 7a,c, it can be seen that the side lobes of the SR image are significantly weakened. Comparing Figure 7b,d, we find that the 2D wave number spectrum of the SR image is broadened, which indicates that the proposed MMW image SR algorithm is effective and reliable.

**Figure 8:**
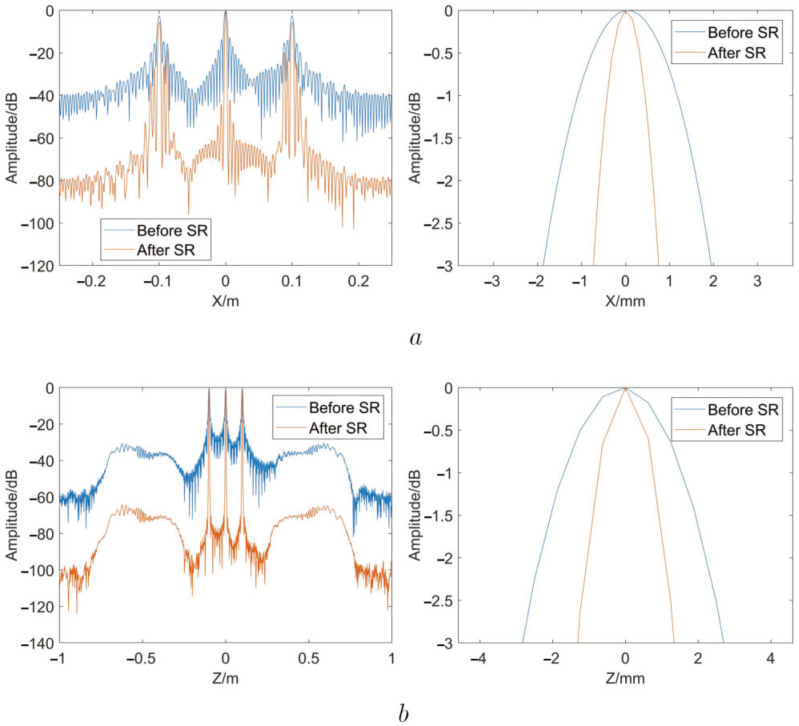
Point target response functions. (**a**) The comparison of point target response functions before and after SR in X dimension at Z = 0 m. (**b**) The comparison of point target response functions before and after super-resolution in Z dimension at X = 0 m.

In order to quantitatively give the results, the point target response functions before and after SR are shown in Figure 8a,b, respectively. From Figure 8a, it can be known that the 3 dB resolution in the X dimension proves improved when its value decreases from 3.9 to 1.8 mm, and the side-lobe amplitude is reduced by about 40 dB. From Figure 8b, it can be seen that the 3 dB resolution in the Z dimension is improved when its value decreases from 5.6 to 2.7 mm, the side-lobe amplitude is reduced by about 40 dB.

The imaging results of these lattice point targets imaging results show that the MMW 3D image SR method described in this paper is effective. The BCS algorithm can be used to significantly expand the wave number spectrum of the 2D image. In addition to improving the main lobe resolution of the targets, the image super-resolution algorithm also significantly reduces the side-lobe amplitude, which indicates that the image resolution and quality are greatly improved.

### 4.2. Measured Super-Resolution Experiment of Human Body Image

**Figure 9:**
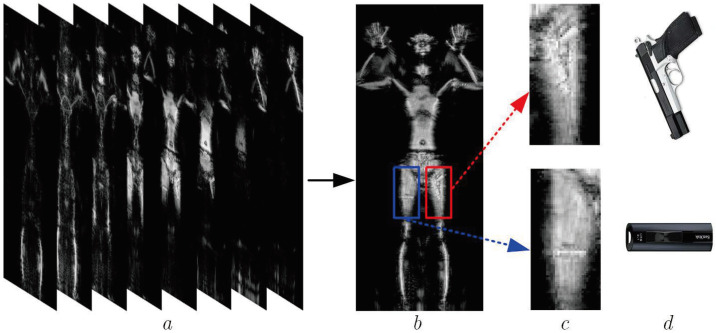
Raw imaging results of measured data. (**a**) Three-dimensional (3D) image of measured data. (**b**) Maximum projection result of 3D image. (**c**) Enlarged MMW image of toy gun and USB disk using linear stretch. (**d**) Optical image of toy gun and USB disk.

In order to further verify the effectiveness and accuracy of this proposed regional image SR method, the MMW 3D imaging system of the human body is applied to collect the echo data of the human body and image it. The parameters of the system are shown in Table 2, and the 3D images are shown in Figure 9.

As shown in Figure 9, the raw imaging results obtained through the MMW holographic imaging system are presented. In the experiment, a metal toy gun and a USB disk as shown in Figure 9d were hidden in the model’s pocket. Figure 9a illustrates the raw MMW 3D image. Its 2D projection image is shown in Figure 9b according to (13). It can be known from Figure 9b that the MMW can penetrate human clothing to image the hidden objects, the toy gun in the red box and the USB disk in the blue box. To get a better look, their enlarged images have been presented, respectively, as shown in Figure 9c. It can be seen from Figure 9c that the resolution of the images is too low to perform threat detection and recognition; thus, the image SR is required.

According to the regional 3D image super-resolution scheme, a pre-detection of suspicious targets is first performed on the 2D projection image of the MMW 3D image to obtain the ROI (as shown in the red box and blue box in Figure 9b). Then, the resolution enhancement is carried out for the useful 2D slice images of the regional 3D image corresponding to the ROI through compressive sensing. Finally, we perform the maximum projection for the regional 3D image with higher resolution to obtain its projection 2D image for threat recognition.

**Figure 10:**
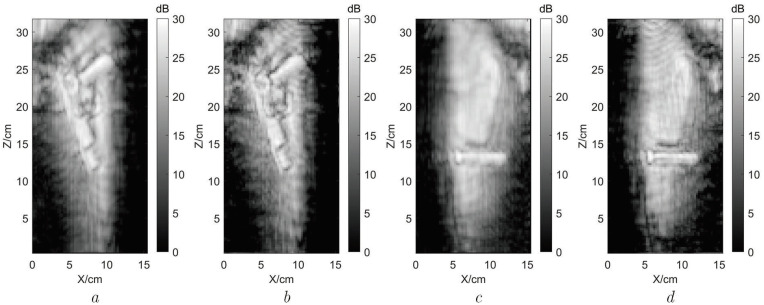
Comparison of image before and after regional image SR. (**a**) Regional enlarged image of toy gun using bilinear interpolation. (**b**) Regional enlarged image of toy gun using regional image SR. (**c**) Regional enlarged image of USB disk using bilinear interpolation. (**d**) Regional enlarged image of USB disk using regional image SR.

The resolution of the system in this experiment is $\delta_{x}\approx 5.5$ mm and $\delta_{z}\approx 5.5$ mm. In this system, the non-idealities of the chirp signal introduce system noise. To obtain better imaging quality, we added a Hamming window to the signal during the imaging process to suppress noise and side lobes, resulting in the error of 0.5 mm between the resolution and its theoretical value. After image super-resolution, the image resolution in the X-Z plane can increase from 5.5 to 4 mm, and the imaging performance is shown in Figure 10.

As shown in Figure 10, the pixel value ranges from 0 to 30dB in each sub-image. Bilinear interpolation is used to enlarge the useful slice images of the two suspicious targets shown in Figure 9c. We operate the maximum projection for the two groups of slice images and present their results in Figure 10a,c. Our proposed method is used to process the three-dimensional images of the two suspicious targets, and the results are shown in Figure 10b,d. According to Figure 10, it is shown that the images obtained through our proposed method are more clear, especially in the edges and details, than the bilinear interpolation images. Therefore, the experiment results show that the regional image SR method is effective and correct in actual MMW image resolution enhancement processing, which is beneficial to the subsequent threat object detection and recognition process.

## 5. Conclusions

Due to the image sparsity of active MMW 3D imaging, this paper studies the method of MMW image SR based on wave number spectrum distribution and compressive sensing algorithm, and it develops a regional 3D image SR scheme to improve threat detection and recognition. First, the working principle and holographic imaging algorithm of the active MMW imaging system are discussed, and the wave number spectrum distribution and image sparsity of MMW 3D images are analyzed. Then, the echo data and 3D imaging of the lattice point targets are simulated according to the active MMW working mode, and the SR image reconstruction results verify the effectiveness and feasibility in image resolution enhancement and image quality improvement. Finally, the correctness of the proposed method in measured image resolution improvement is demonstrated by the human body imaging experiment.

## Figures and Tables

**Table 1:** Simulation parameter settings of the MMW 3D imaging system.

Item	Value
Operation frequency ($f_{r}$)	32–38 GHz
Beam width of pitch	$60^{\circ }$
Number of sensors	400
Interval of sensors	5 mm
Beam width of azimuth	$60^{\circ }$
Radius of gyration	0.7 m
Azimuth sampling interval	$0.25^{\circ }$

**Table 2:** Operating parameters of the MMW 3D imaging system.

Item	Value
Operation waveband	Ka
System bandwidth	5 GHz
Radius of gyration	0.7 m
Azimuth sampling interval	$0.4^{\circ }$
Azimuth resolution ($\delta_{x}$)	5 mm
Range resolution ($\delta_{y}$)	30 mm
Vertical resolution ($\delta_{z}$)	5 mm

## Data Availability

Not applicable.

## Abbreviations

The following abbreviations are used in this manuscript:

MMW	Millimeter-wave
SAR	Synthetic aperture radar
ROI	Region of interest
CS	Compressive sensing
BCS	Bayesian compressive sensing
FFT	Fourier transform
2D	Two-dimensional
3D	Three-dimensional
SR	Super-resolution
PSNR	Peak signal-to-noise ratio
POSP	Principle of stationary phase
SM	Stolt Mapping
BP	Back projection
MAP	Maximum a posterior
CGA	Conjugate gradient algorithm
